# Altered Innate Immune and Glial Cell Responses to Inflammatory Stimuli in Amyloid Precursor Protein Knockout Mice

**DOI:** 10.1371/journal.pone.0140210

**Published:** 2015-10-08

**Authors:** Anna Carrano, Pritam Das

**Affiliations:** Department of Neuroscience, Mayo Clinic Florida, Jacksonville, Florida, United States of America; Indiana School of Medicine, UNITED STATES

## Abstract

Amyloid precursor protein (APP) and its cleaved products have been reported to have important functions in CNS health, including in memory and synapse formation, cell survival and neuroprotection. Furthermore APP and its cleaved products have been shown to be transiently increased in response to various CNS stressors, suggesting a role in response to acute cellular injury. In an attempt to further understand the function of APP in response to CNS injury, we have used intracranial LPS injection as an inflammatory injury model in APP knock out mice (APPKO). Our data show that innate immune responses to LPS injection is significantly blunted in APPKO mice compared to APP sufficient wild type (BL6) mice. Morphologically, glial cells in APPKO mice appear less reactive, with shorter ramified processes and smaller cell bodies in response to LPS. Additionally, quantitative RT-PCR analysis for several glia markers and innate immune cytokine levels (e.g. TNFα, IL-6, IL-1β and IL-10) showed significantly reduced expression levels in LPS injected APPKO mice. In vitro cell culture assays confirmed this attenuated response to LPS stimulation by primary microglial cells isolated from APPKO mice. Our data suggests that APP full length protein and/or its cleaved products are necessary to mount a complete and effective innate immune cell response to inflammatory injury.

## Introduction

Alzheimer disease (AD) is the most common cause of dementia for which an effective treatment is not available yet. The most widely accepted hypothesis states that AD is initially triggered by the abnormal accumulation of amyloid β-peptide (Aβ) in the brain, which in turn initiates a pathogenic cascade that ultimately leads to neuronal death and dementia [1]. Aββ is cleaved from a long membrane-bound precursor, the amyloid precursor protein (APP), by two consecutive cleavages. β- and γ-secretases are the enzymes that liberate the N and C termini of Aβ, respectively [2]. Although much is known about Aβ pathophysiology, the normal physiological functions of APP and its cleaved fragments are not well understood, particularly in response to brain aging and inflammation. Evidence to suggest that APP and its cleavage fragments may support a trophic function of APP in neurons and synaptic activities [3], but very little is known about the role of APP/APP fragments in the innate immune response to acute CNS injury. Furthermore, it has been reported that both APP and its cleaved products, are transiently increased in response to various CNS stresses, although the reasons for this up-regulation is not well understood [4–7].

In an attempt to further understand the role of APP in response to CNS injury, we have performed experiments using intracranial LPS injection as an inflammatory injury model in APPKO mice.

Our data indicates that mice lacking APP present with an “altered” innate immune response to LPS-induced brain inflammation. Microglial cells and astrocytes in APPKO mice appear less reactive; these mice have reduced expression of glial markers and reduced expression of several inflammatory innate immune cytokines following LPS stimulation. Based on these findings, we propose that APP and/or its cleaved fragments play an important role on glial cell activation and the innate immune response to CNS injury. Furthermore, these results suggest that APP may also interact, either directly or indirectly, in the LPS-TLR signaling pathways, supporting a novel function of APP in response to inflammatory stimuli.

## Material and Methods

### Mice

APP -/- mice were maintained and genotyped as described previously [8], with both the APP+/+ and APP-/- mice on the same background strain, C57BL6J and were purchased from Jackson Laboratories. All animal husbandry procedures performed were approved by the Mayo Clinic Institutional Animal Care and Use Committee in accordance with National Institutes of Health guidelines. All animals were housed three to five to a cage and maintained on *ad libitum* food and water with a 12h light/dark cycle and were used for study between 3 and 9 months of age.

### Intrahippocampal LPS injections

Mice were anesthetized using isoflurane and immobilized in a stereotaxic apparatus. A 2 μl injection of 4 μμg/μl LPS (Salmonella abortus equi; Sigma, St. Louis, MO) was delivered over a two min period into both the hippocampi (coordinates from bregma: −2 mm posterior, −/+ 2 mm lateral, and −2.0 mm ventral). The incision was closed with surgical glue, isoflurane was discontinued, and the animal revived under a heating lamp. All mice completely recovered within 5 min. Animals were singly housed for the post-treatment survival period under standard vivarium conditions. We used *n = 4–8* mice/group for each condition. Mice were sacrificed at 1 or 3 days post-surgery. Right brain hemispheres were fixed in 4% paraformaldehyde for histological analysis. Left brain hemispheres were dissected in hippocampus, cortex, midbrain and cerebellum and kept frozen at −80°C until further analysis.

### Immunohistochemistry

Paraffin embedded sections were stained for microglial marker Ionized calcium-binding adaptor molecule 1 (Iba-1, 1:500; Wako Chemicals) antibody and visualized through the Dako Envision Plus visualization system [9]. Immunohistochemically stained sections for Iba-1 were captured using the ScanScope XT image scanner (Aperio Technologies). Microglial cell counts and morpholological analysis (microglial process length and cell body size) in hippocampus were quantified using MetaMorph Microscopy Automation & Image Analysis Software (Molecular Devices).

### Primary Microglia

Mixed glia cultures were derived from postnatal day1-3 C57BL/6J and APP-/- mice. Briefly, cerebral cortices were isolated, meninges were removed, and tissue was minced in the presence of DNAse I (50 μg/ml, Sigma). Cell suspension was filtered with a 40 μm cell strainer and plated in 75-cm^2^ flasks. Cells were cultured in Dulbecco’s modified Eagle’s medium (DMEM) media (Gibco) containing 10% FBS (Gibco), 1% pen-strep (Gibco), 25 ng/ml mGM-CSF (R&D Systems). Media was replaced every 5 days. At approximately 14 and 21 days microglia were harvested by rapid shaking for 30 minutes.

Isolated microglia cells were cultured for 24 hours in DMEM+1% pen-strep followed by treatment with 100 ng/ml LPS for 4 hours. Cells were collected and RNA was extracted from using TRIzol (Invitrogen).

### Western Blot

Hippocampi were dissected and snap frozen for subsequent analysis. Brain tissue was sequentially extracted in Tris-buffered saline (TBS), TBS buffer containing 1% Triton X-100 (TBSx) and 5M guanidine in 50 mm Tris-HCl, pH 8.0 (GN-HCL) as previously described [10]. Membranes containing TBSx extracted protein samples separated on Bis-Tris 12% XT gels (Bio-Rad, Hercules, CA, USA) were probed with the antibody CT20 (anti-APP C-terminal 20 amino acid; 1:1000), as previously described [9].

### Quantitative RT (qRT)-PCR

Total RNA from mice hippocampus was extracted using the RNeasy mini kit (Qiagen) following the manufacturer’s protocol. Total RNA was dissolved in nuclease-free water and stored at −80°C. Reverse transcription was performed using Superscript III (Invitrogen) using Mastercycler pro (Eppendorf) and the reaction mix was subjected to qRT-PCR using iQ SYBR Green Supermix (Bio-rad) to detect the amplification products. Relative quantification of mRNA expression was calculated by the ΔC_T_ method after adjusting the levels to the corresponding internal GAPDH control for each sample.

The sequences of primers used to amplify target genes by qRT-PCR were as follows:

GAPDH 5’- AGGTCGGTGTGAACGGATTTG-3’ 5’- TGTAGACCATGTAGTTGAGGTCA-3’;

Iba-1 5’-CTTGAAGCGAATGCTGGAGAA-3’ 5’-GGCAGCTCGGAGATAGCTTT-3;

IL-1β 5’-CTGTGACTCATGGGATGATGATG-3’ 5’-GCCTGTAGTGCAGTTGTCTAAT-3’;

IL-6 5’- TCTATACCACTTCACAAGTCGGA-3’ 5’-GAATTGCCATTGCACAACTCTTT-3’;

IL-10 5’- ACAGCCGGGAAGACAATAACT-3’ 5’-GCAGCTCTAGGAGCATGTGG-3’;

TNF-α 5’- CAGGCGGTGCCTATGTCTC-3’ 5’-CGATCACCCCGAAGTTCAGTAG-3’;

TGF-β 5’-TCGACATGGATCAGTTTATGCG-3’ 5’-CCCTGGTACTGTTGTAGATGGA-3’;

CD11b 5’- GTGTGACTACAGCACAAGCCG-3’ 5’-CCCAAGGACATATTCACAGCCT-3’;

CX3CR1 5’- ACCGGTACCTTGCCATCGT-3’ 5’-ACACCGTGCTGCACTGTCC-3’;

Trem2 5’- GCCTTCCTGAAGAAGCGGAA-3’ 5’-GAGTGATGGTGACGGTTCCA-3’;

DAP12 5’- GATGCTTACCTGGGTTATGCTTCT-3’ 5’-CCGAGGTGCTCCTAAAACCA-3’;

TLR4 5’-GCTTACACCACCTCTCAAACTT-3’ 5’-AACTTCCTGGGGAAAAACTCTG-3’;

P2ry12 5’-CACGGATTCCCTACACCCTG-3’ 5’-GGGTGCTCTCCTTCACGTAG-3’;

Hexb 5’-ACTCCAAGATTATGGCCTCGAGCA-3’ 5’-GCTATTCCACGGCTGACCATTCT-3’.

Quantified expression levels are reported in Table A in S1 File and Table B in S1 File.

### Statistical analysis

Two-way ANOVA with *post hoc* Tukey’s multiple-comparison test or 2-tailed Student’s *t* test was used for statistical comparison (GraphPad Prism 6, GraphPad Software).

## Results

### 1. Glial cell response to LPS-induced inflammation in 3 months old APPKO mice.

**Fig 1:**
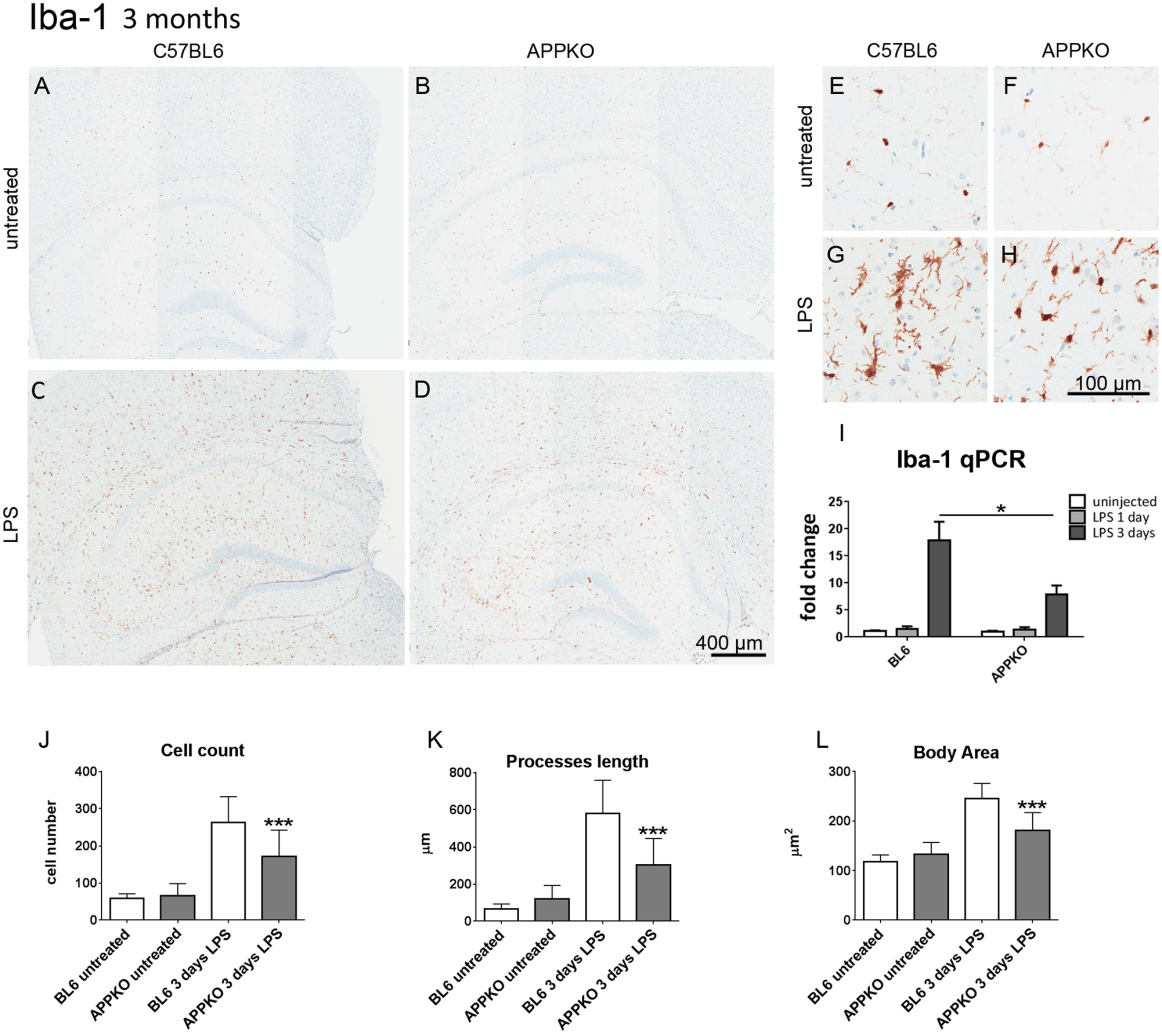
Microglia response to LPS-induced inflammation is hampered in 3 month old APPKO mice. (A-H) Representative brain sections immuno-stained with anti-Iba1 in hippocampus of 3 month old mice (A-D), higher magnification shown in (E-H). Iba-1 expression in microglial cells was increased 3 days after LPS injection (C,D,G,H) in both APPKO and wild type C57BL6 mice. However, this increase in microglial Iba1 immuno-staining was blunted in APPKO mice (D, H) compared to APP sufficient C57BL6 wild type mice (C,G). Quantitative RT-PCR for Iba-1 mRNA levels shows significant reductions in Iba-1 transcripts in APPKO mice 3 days after LPS injection (I). Detailed cell count and morphological analysis show that the number of Iba-1 positive microglia cells in the hippocampus were less in APPKO mice (J), the microglial cells present with shorter processes in APPKO mice (K) and the microglial cell body area was smaller in APPKO mice (L) in response to LPS stimulation. * p<0.05, *** p<0.001 APPKO vs C57BL6.

APPKO and wild type BL6 mice following hippocampal LPS injections were sacrificed at day 1 and 3 post surgery and analyzed for immuno-histochemical -staining of microglial marker Iba-1. (Fig 1A–1H) shows that LPS stimulation induces microglia activation and proliferation at 3 days post injection in both 3 months old APPKO mice and wild type BL6 mice. This microglia response appears to be severely compromised in APPKO mice. Iba-1-positive microglia cell number was significantly reduced in APPKO (↓ 34.6%) compared to wild type BL6 mice (Fig 1J). APPKO microglia processes are shorter and less ramified (↓47.8%) and cell body size was smaller (↓26.2%) compared to BL6 controls (Fig 1K and 1L). However this microglia phenotype, as measured by cell count, process length and body size, was not significantly different in naïve untreated APPKO mice compared to naïve wild type BL6 mice (Fig 1J–1L).

**Fig 2:**
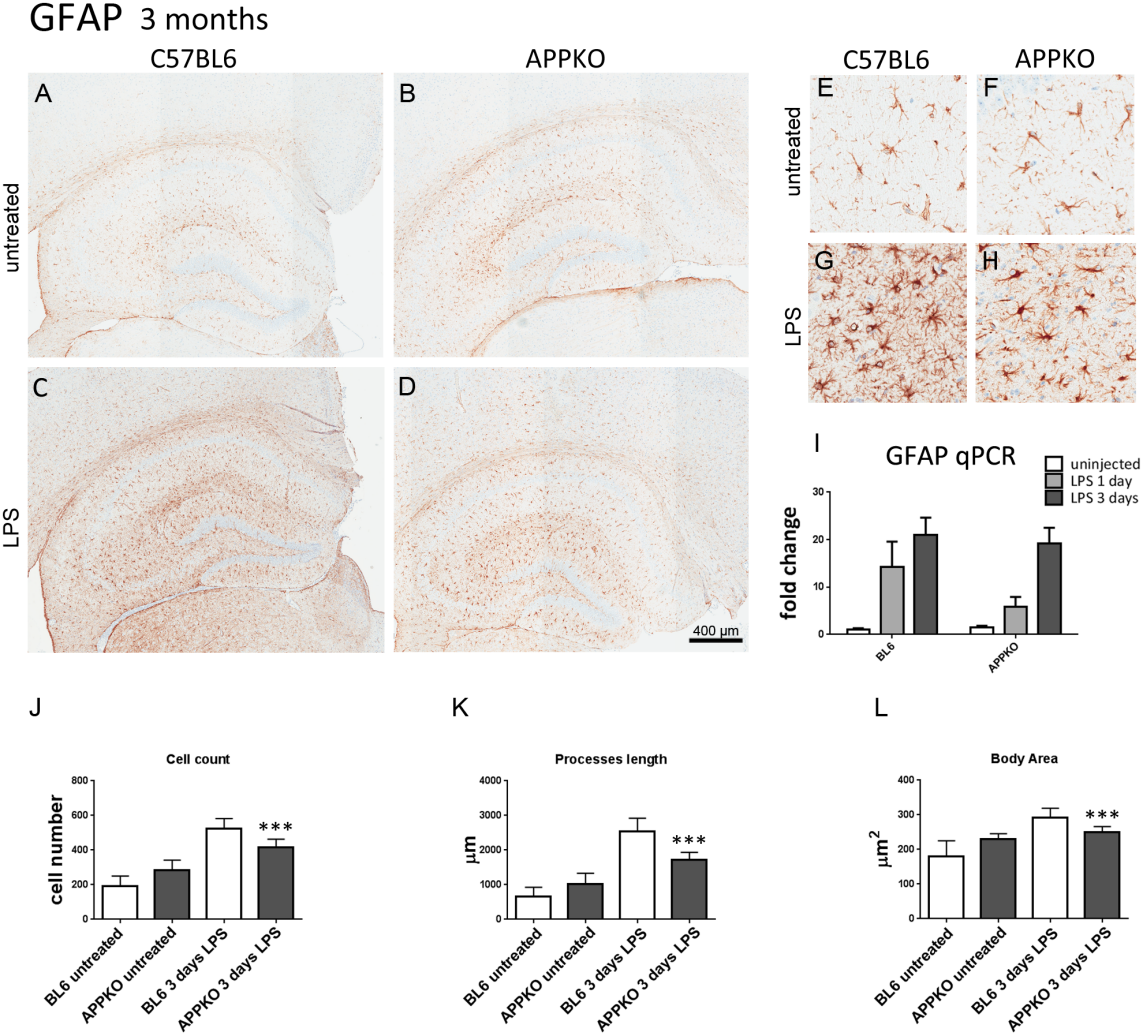
Astrocytes response to LPS-induced inflammation is hampered in 3 month old APPKO mice. (A-H) Representative brain sections immuno-stained with anti-GFAP in hippocampus of 3 month old mice (A-D), higher magnification shown in (E-H). GFAP expression in astrocytic cells was increased 3 days after LPS injection (C,D,G,H) in both APPKO and wild type C57BL6 mice. However, this increase in GFAP immuno-staining was blunted in APPKO mice (D, H) compared to APP sufficient C57BL6 wild type mice (C,G). Quantitative RT-PCR for GFAP mRNA levels shows significant reductions in GFAP transcripts in APPKO mice 3 days after LPS injection (I). Detailed cell count and morphological analysis show that the numbers of GFAP positive astrocytes in the hippocampus were less in APPKO mice compared to BL6 mice (J), astrocytic cells present with shorter processes in APPKO mice (K) and the cell body area is smaller in APPKO mice (L) in response to LPS stimulation. *** p<0.001 APPKO vs C57BL6.

Quantitative RT-PCR shows a significant reduction of Iba-1 mRNA transcripts in the hippocampus of APPKO mice compared to BL6 mice in response to LPS injection only at 3 days post-surgery (Fig 1I). No significant changes in Iba-1 mRNA levels were observed at 1 day post LPS injection. In the 3 month old cohorts, reactive astrogliosis was induced by LPS injection both in the BL6 as well as APPKO mice (Fig 2A–2H). Astrocytes were visualized and quantified by immunostaining using anti- GFAP antibody. Similar to microglia cells, astrocyte cell counts were reduced (↓20.7%) (Fig 2J), astrocytes were smaller in size (↓14.4% body area, Fig 2L) and had shorter processes (↓32.6%) in APPKO mice following LPS stimulation (Fig 2K). Despite these morphological differences, mRNA levels of astrocytic marker GFAP was not significantly different between BL6 and APPKO mice, after LPS injection (Fig 2I).

### 2. Glial cell response to LPS-induced inflammation in 9 months old APPKO mice

**Fig 3:**
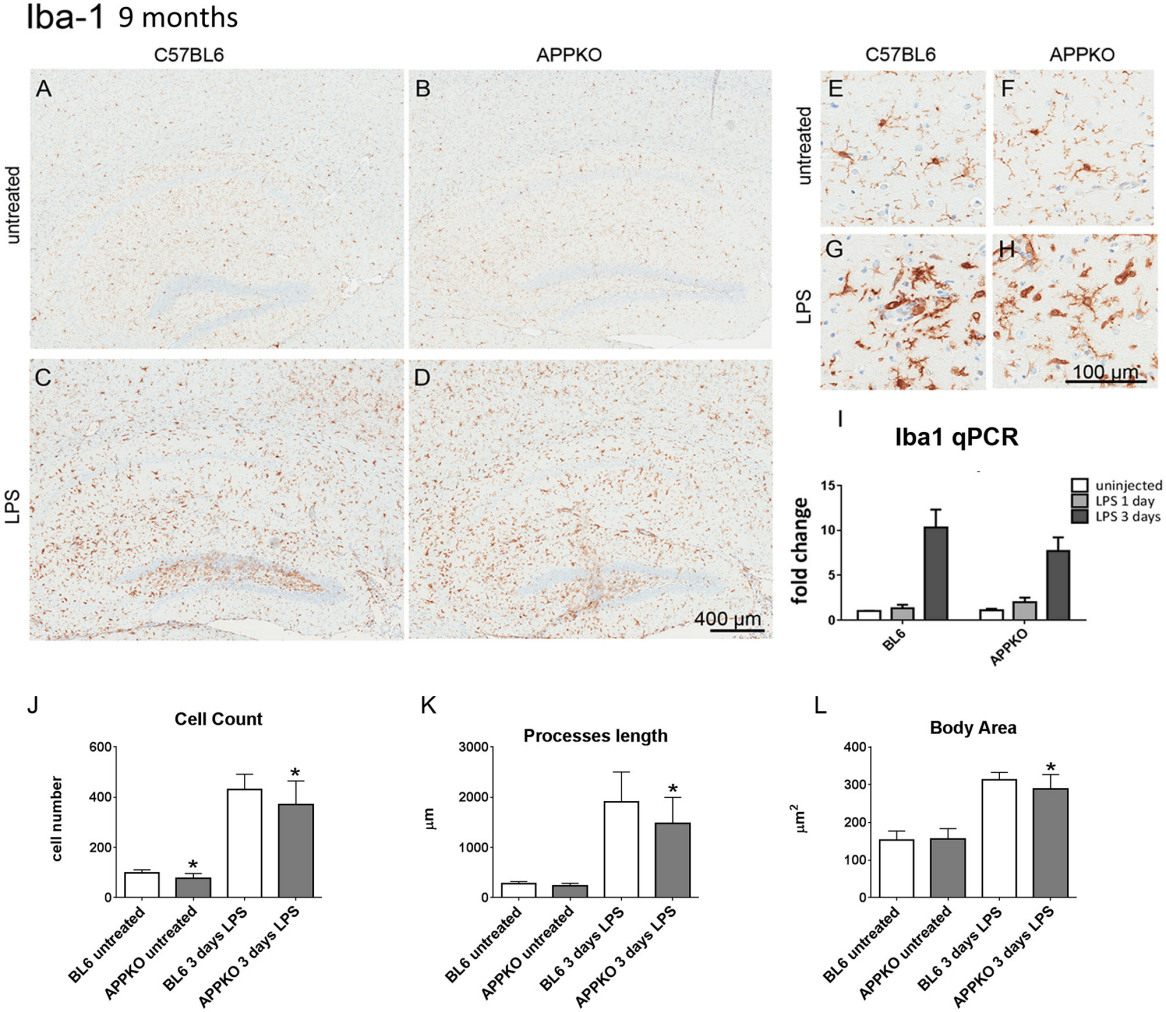
Microglia response to LPS-induced inflammation is hampered in 9 months old APPKO mice. (A-H) Representative brain sections immuno-stained with anti-Iba-1 in hippocampus of 9 month old mice (A-D), higher magnification shown in (E-H). Iba-1 expression in microglial cells was increased 3 days after LPS injection (C,D,G,H) in both APPKO and wild type C57BL6 mice. However, this increase in microglial Iba1 immuno-staining was blunted in APPKO mice (D, H) compared to APP sufficient C57BL6 wild type mice (C,G). qPCR for Iba-1 mRNA levels show reductions in Iba-1 transcripts in APPKO mice 3 days after LPS injection (I). Detailed cell count and morphological analysis showed that the numbers of Iba-1 positive microglia in the hippocampus were less in APPKO mice (J), the microglial cells present with shorter processes in APPKO mice (K) and the microglial cell body area was smaller in APPKO mice (L) in response to LPS stimulation. * p<0.05 APPKO vs C57BL6.

(Fig 3A–3H) shows that LPS stimulation-induces microglia activation and proliferation in both APPKO and wild type BL6 mice in 9 months old mice at 3 days post LPS injection. Similarly to the younger cohort, in the 9 months old cohort, Iba-1 positive cell number (↓14%), cell size (↓7.8%), and processes length (↓22.8%) were also reduced in the APPKO mice compared to BL6 mice following LPS injection (Fig 3J–3L). Nine months old untreated APPKO mice also presented with significantly reduced iba-1 positive cell numbers (↓20.7%, Fig 3J). Quantitative RT-PCR showed a slight reduction, although not significant, in Iba-1 mRNA transcripts in APPKO mice compared to BL6 mice in response to LPS injection at 3 days post-injection whereas no significant changes in Iba-1 mRNA levels were observed at 1 day post LPS injection (Fig 3I).

**Fig 4:**
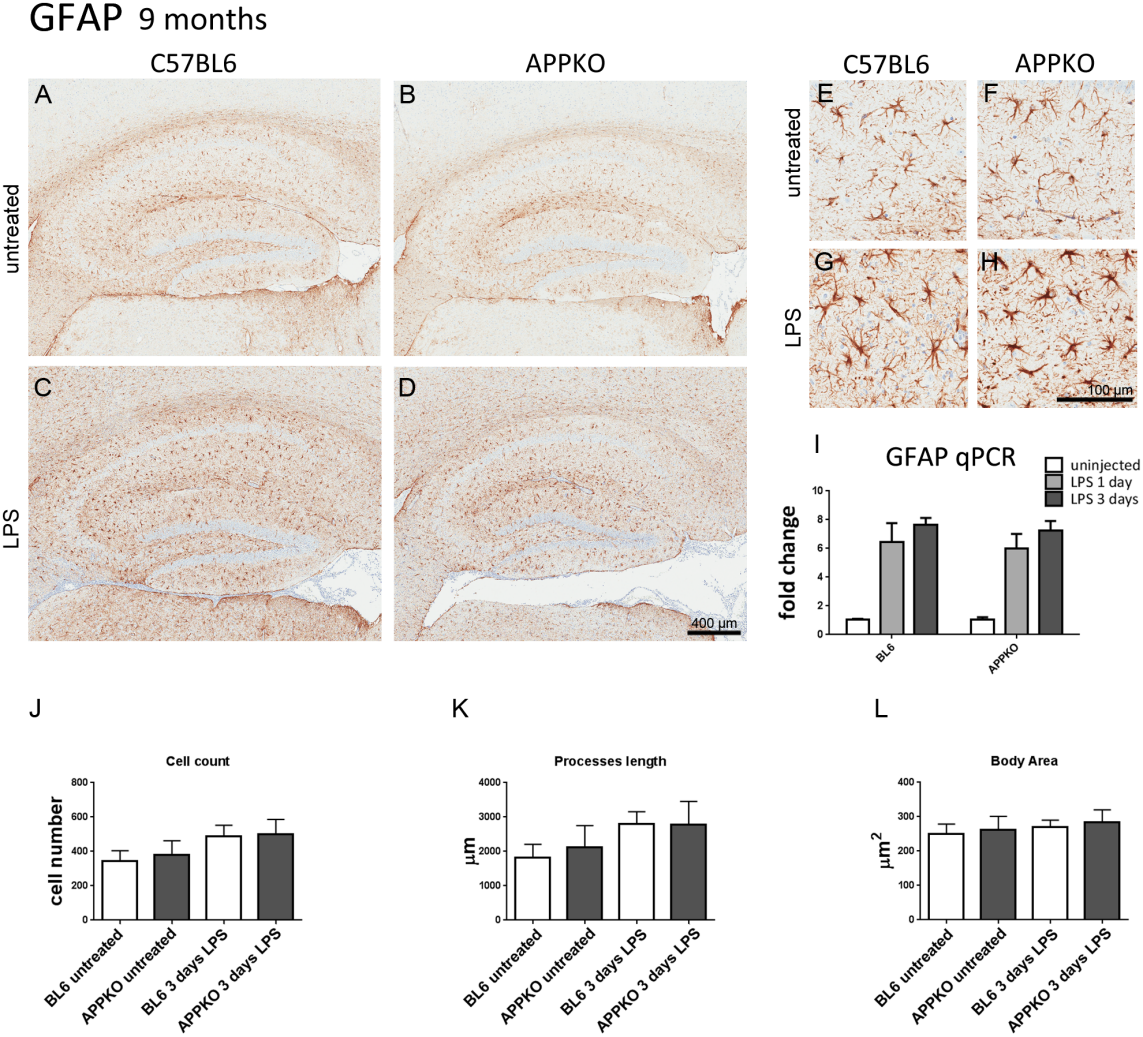
Astrocytes response to LPS-induced inflammation in 9 months old APPKO mice. (A-H) Representative brain sections immuno-stained with anti-GFAP in hippocampus of 9 month old mice (A-D), higher magnification shown in (E-H). GFAP expression in astrocytes was increased 3 days after LPS injection (C,D,G,H) in both APPKO and wild type C57BL6 mice. (I) qPCR analysis showed increased expression of GFAP mRNA levels in both APPKO mice and BL6 mice following LPS stimulation, however, there were no significant differences in GFAP mRNA levels between APPKO mice compared to BL6 mice. Detailed cell count and morphological analysis showed that the numbers of GFAP positive cells in the hippocampus were comparable in APPKO mice and BL6 (J), there were no significant differences in cell processes length (K) nor cell body area (L) in APPKO mice compared to BL6 in response to LPS stimulation.

In the 9 month old cohorts, detailed morphological analysis of reactive astrogliosis measured by immunostaining using anti-GFAP antibody (Fig 4A–4H and 4J–4L) did not show any significant differences between BL6 and APPKO mice in response to LPS injection. Similarly, qPCR analysis showed no significant differences in GFAP mRNA levels between APPKO mice compared to BL6 mice. (Fig 4I).

### 3. Microglial markers profile in APPKO mice after LPS treatment

**Fig 5:**
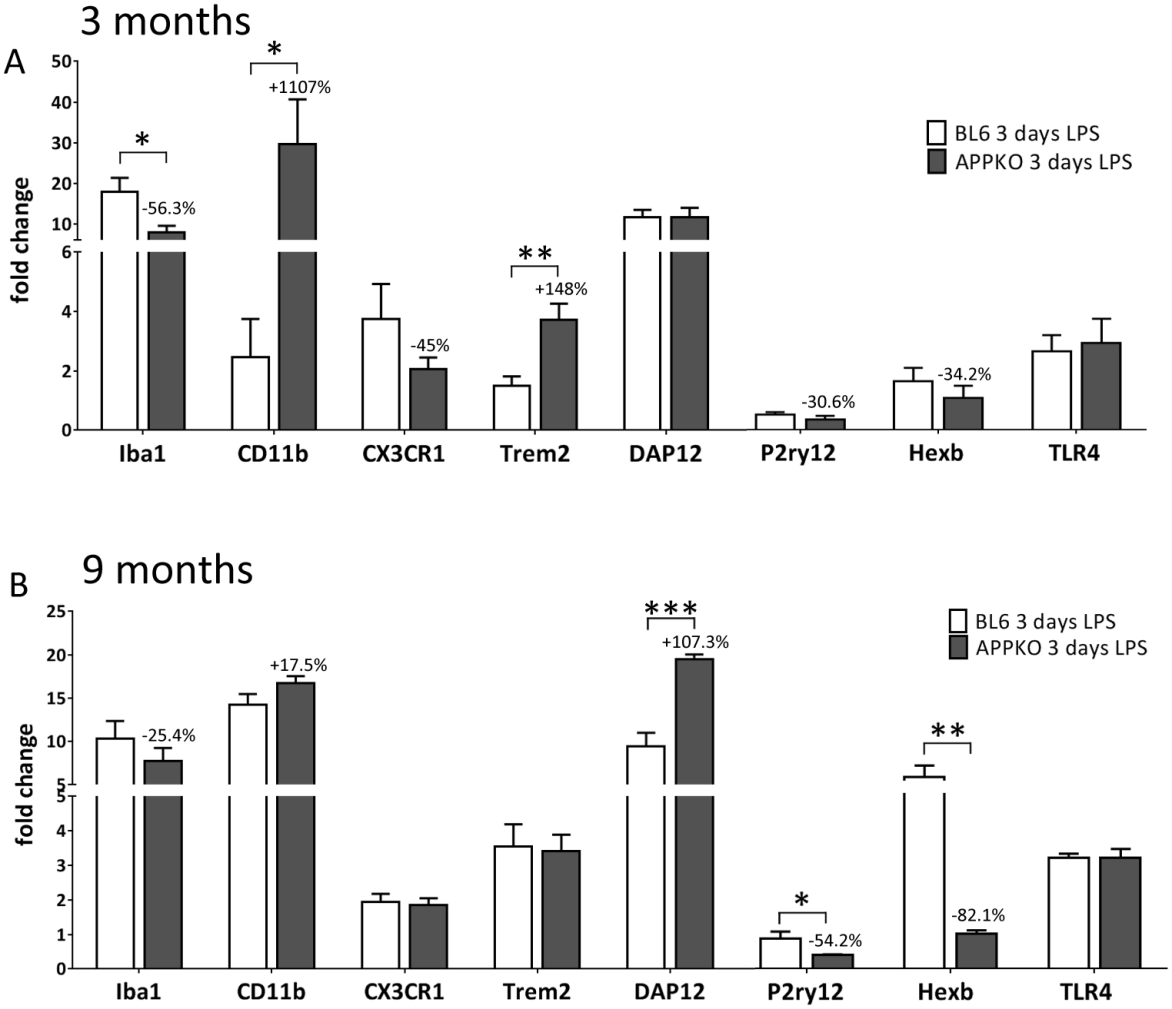
Microglial markers profile in APPKO mice after LPS treatment Quantitative RT-PCR analysis of hippocampal mRNA levels from 3-month old (A) and 9 months old mice (B) showed altered expression pattern of several microglia markers in APPKO mice compared to C57BL6 wild type mice in response to hippocampal LPS injection at 3 days after surgery. Data is represented as fold change over untreated control mice. *p<0.05, ** p<0.01, *** p<0.001 (C57BL6 vs APPKO).

Our immunohistochemical analysis revealed a defective glial response in APPKO mice, where microglia activation appeared particularly compromised. To further validate our data, we measured the expression levels of several microglial markers by quantitative real-time PCR in APPKO mice vs. BL6 mice following LPS injection (Fig 5). In addition to Iba-1, we also assessed expression for CD11b, a microglial pattern recognition and phagocytic receptor [11], CX3CR1 (chemokine fractalkine receptor) [12], Trem2 and its functional partner protein DAP12 [12], the microglia specific purinergic receptor P2ry12 [13, 14], the enzyme hexosaminidase B (Hexb) and toll-like receptor 4 (TLR4).

In the 3 month old cohorts, expression of Iba1, CX3CR1, P2ry12 and Hexb was lower, in APPKO mice compared to BL6 after LPS injection (Fig 5A). On the other hand, CD11b and Trem2 mRNA levels were significantly increased in APPKO mice compared to BL6 mice (Fig 5A). We did not observe any difference in DAP12 and TLR4 expression between APPKO and wild type mice (Fig 5A).

In the 9 month old cohorts, mRNA expression of only P2ry12 and Hexb, were significantly reduced in APPKO mice compared to BL6 after LPS injection, whereas DAP12 was the only microglia marker that showed an increased upregulation in APPKO mice. Expression levels of other microglial markers, including Iba1, s CD11b, CX3CR1, Trem2 and TLR4, were not significantly different between APPKO and BL6 mice (Fig 5B).

### 4. Inflammatory cytokine levels are reduced in APPKO mice after LPS treatment

**Fig 6:**
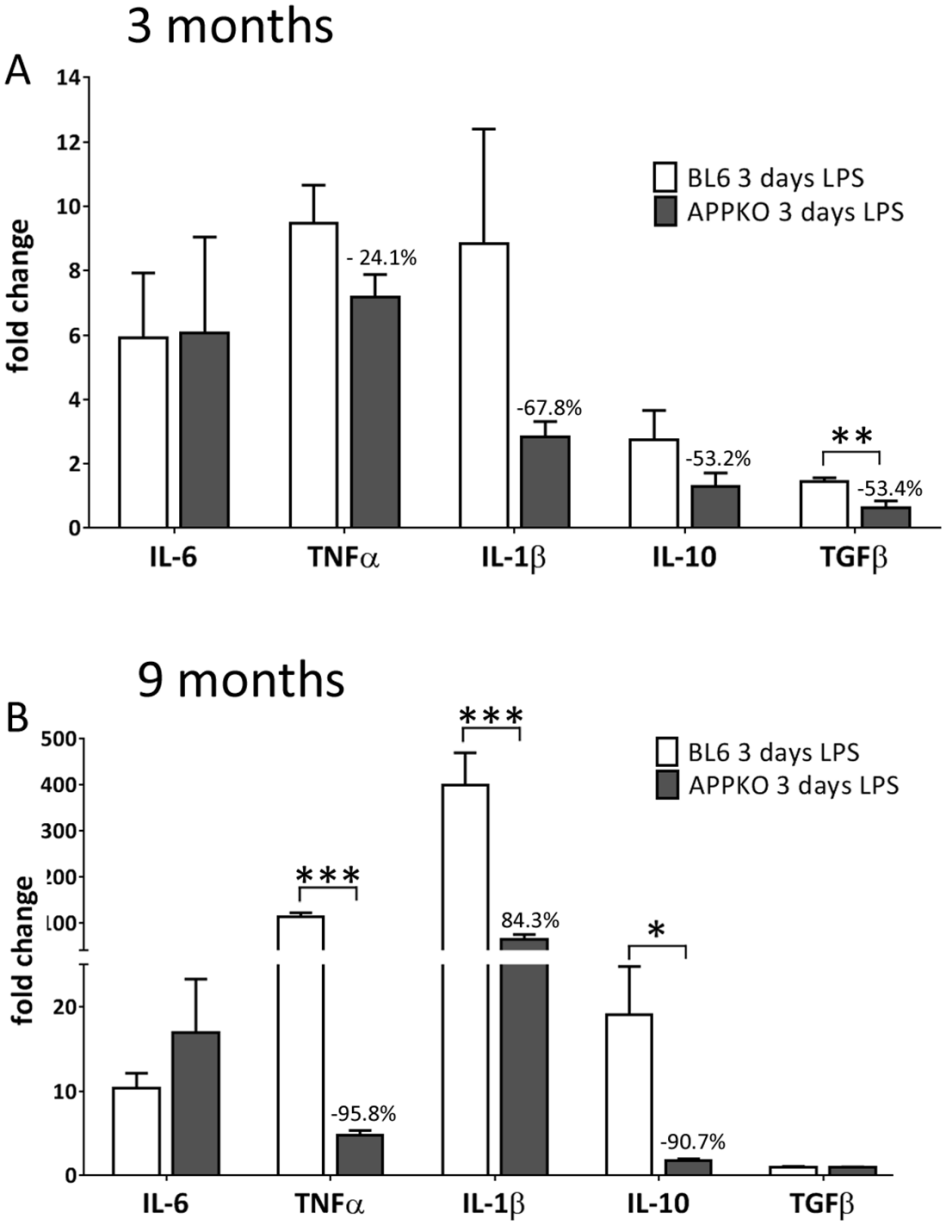
Reduced inflammatory cytokine levels in APPKO mice after LPS treatment. Quantitative RT-PCR analysis of hippocampal mRNA levels from 3 months old (A) and 9 months old (B) mice showed reduced expression pattern of several inflammatory cytokines (TNF-α, IL-1β, Il-10 and TGFβ) in response to LPS injection. Data is represented as fold change over untreated control mice. *p<0.05, ** p<0.01, *** p<0.001 (C57BL6 vs APPKO).

In vivo exposure to LPS activates parenchymal microglia and astrocytes, and induces cytokine and chemokine production in the brain [15]. By quantitative RT-PCR we measured the expression on IL-6, TNF-α, IL-1β, Il-10 and TGFβ in the hippocampus of LPS treated mice using both 3 and 9 month old cohort. As shown in Fig 6A, majority of the of the cytokines we measured were lower in 3 month old APPKO mice compared to cytokine levels in BL6 mice wild type at 3 days post LPS injection, except for IL-6, which did not show any significant differences.

In the 9 month old cohorts, the expression of inflammatory cytokines was more drastically reduced in APPKO mice compared to BL6 following LPS injection with >90% reductions in TNFα, IL-1β and IL-10 (Fig 6B).

### 5. Synaptic markers expression in APPKO mice after LPS treatment

**Fig 7:**
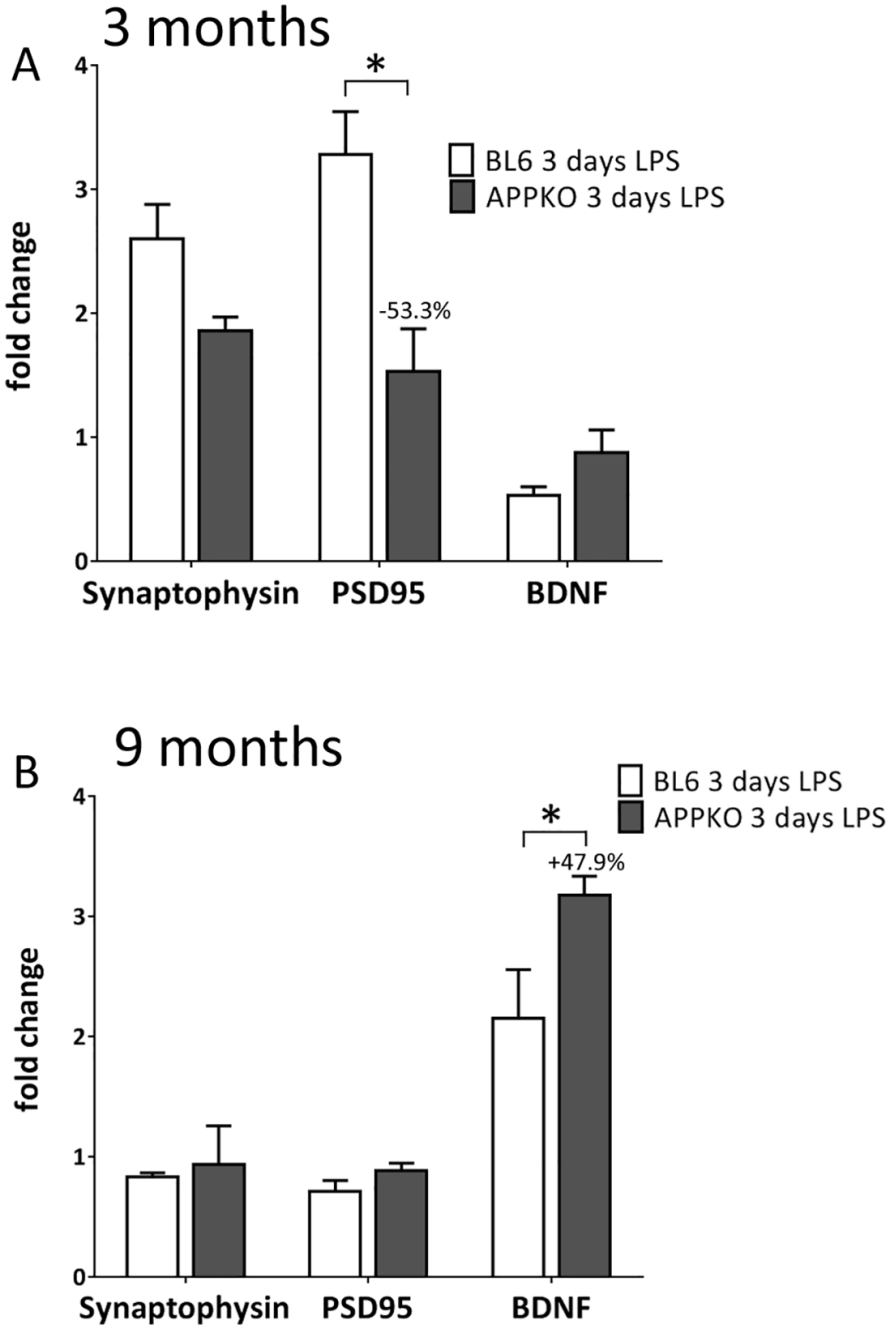
Changes in neuronal markers in APPKO mice after LPS treatment. Quantitative RT-PCR analysis of hippocampal mRNA levels of neuronal markers (synaptophysin, PSD-95 and BDNF) in 3 months old (A) and 9-months old (B) APPKO mice compared to C57BL6 wild type mice in response to LPS injection. Data is represented as fold change over untreated control mice. *p<0.05, ** p<0.01, *** p<0.001 (C57BL6 vs APPKO).

Neuronal and synaptic alterations can occur as a result of neuroinflammation and glia activation in the CNS, therefore we measured changes in synaptic proteins synaptophysin and PSD-95, and brain-derived neurotrophic factor (BDNF) by qRT-PCR in the hippocampus of LPS treated mice. In 3 months old mice, both synaptophysin and PSD-95 mRNA levels were increased in BL6 mice after LPS exposure, probably as a brain repair mechanism following injury. This upregulation, however, was lower in APPKO mice (Synaptophysin ↓28.6% and PSD-95 ↓53.3%). On the other hand, BDNF was downregulated upon LPS treatment in BL6 mice (↓46.5% vs control), whereas its expression is relatively unchanged in APPKO mice (Fig 7A).

Messenger RNA levels of synaptophysin and PSD-95 remain unchanged upon LPS treatment in the 9 months old mice compared to untreated controls (Fig 7B). However, we observed an increase in BDNF levels in both APPKO and BL6 mice following LPS injection compared to untreated controls (Fig 7B).

### 6. Microglial cells response to LPS is impaired in the absence of APP *in vitro*

**Fig 8:**
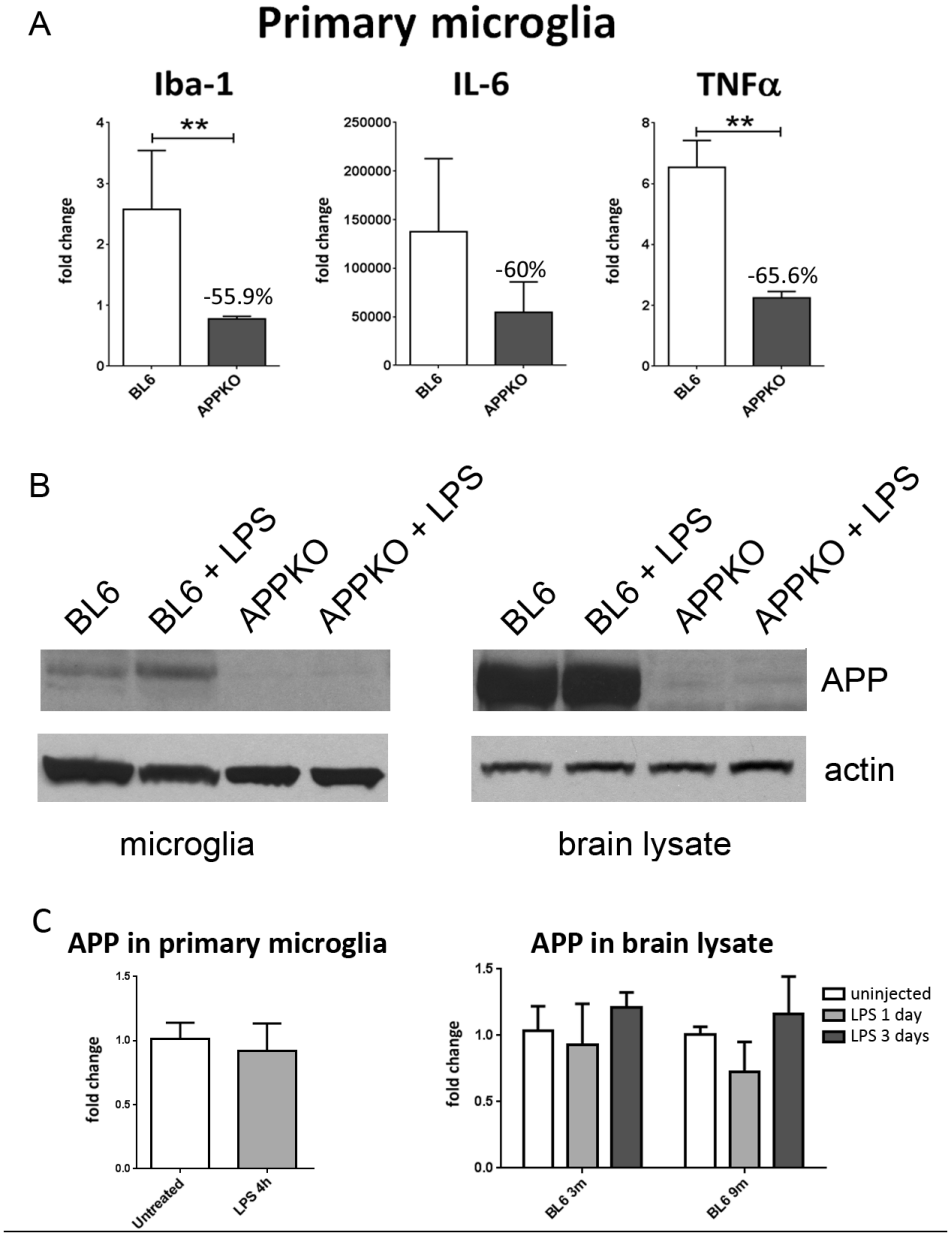
Lack of APP reduces the microglial response *in vitro*. (A) Primary microglia cells isolated from APPKO mice, challenged with LPS for 4 hours expressed lower mRNA levels of Iba-1, IL-6 and TNF-α compared to BL6 cells. (B) Representative anti-APP CT20 immunoblot confirmed the lack of APP protein both in primary microglial cells and brain lysates isolated from APPKO mice. (C) qPCR for APP mRNA showed no significant differences following LPS treatment *in vitro* and *in vivo*. ** p-value<0.01.

In order to confirm our *in vivo* data, primary microglia cells were isolated from BL6 and APPKO neonatal brains and treated with LPS *in vitro*. Primary microglia cells isolated from APPKO mice, challenged with LPS for 4 hours, showed significant decreased in mRNA levels of Iba-1, IL-6 and TNF-α compared to microglial cells from wild type BL6 mice (Fig 8A), supporting an attenuated microglial cell response to LPS in APPKO microglia.

To confirm absence of APP expression in APPKO mice, we performed a western blot immunoassay (anti-APP CT20 antibody), using lysates from both APPKO primary microglial cells and brain tissue. As expected, APP expression is absent in primary microglial cells and brain tissue from APPKO mice whereas BL6 mice show APP expression (Fig 8B). Although previous reports [4–6] have shown a transient increase in APP expression and processing due to injury, in our experimental conditions, we do not see any significant changes in APP mRNA levels after LPS stimulation, both *in vivo* and *in vitro* (Fig 8C).

## Discussion

Prior studies investigating the physiological functions of APP and its cleavage fragments suggest that it plays critical roles in many CNS cellular functions, including synaptogenesis, synaptic plasticity, memory, neurogenesis, and neuroprotection [3].

Furthermore, APP and its cleaved products have been shown to be upregulated both in neurons and glial cells, in response to injury, including ischemia [16] and various other brain injury models [4–6], supporting a role of APP as a stress response protein.

To further understand the role of APP in the context of inflammatory injury, we used lipopolysaccharide (LPS) intracranial injections as a model for acute neuroinflammation and compared the innate immune responses in the brains of APP deficient (APPKO) mice vs. APP sufficient wild type strain C57BL6J (BL6) mice. As readout for the LPS response in these mice, we used both immuno-histological and quantitative RT-PCR analysis, to measure the glial cell responses and the expression of LPS-induced inflammatory cytokines. Using Iba1 as a marker for microglial cells, our immunohistochemical analysis shows that microglial cells from LPS challenged APPKO mice present with a significantly less reactive phenotype, morphologically characterized by reduced body size and shorter process length. Similarly, using GFAP as a marker for astrocytes, we show that astrocytes from LPS challenged APPKO mice also present with a less reactive phenotype, although these differences were not as dramatic as those seen in the microglial cells.

We then measured the mRNA expression profiles of several microglial specific markers/receptors including CD11b, CX3CR1, Trem2, DAP12, P2ry12, Hexb and TLR4 in an attempt to compare changes in the “microglia signature” following LPS challenge in APPKO vs. to wild type B6 mice. Recent studies have identified several of these microglia specific markers/receptors as a measure of brain homeostasis and changes in microglial phenotypes during aging and neurodegenerative disease [13, 14]. Our data reveal an altered “microglia signature” in LPS challenged APPKO mice compared to BL6 mice. RT-PCR analysis of mRNA levels from APPKO mice consistently showed reduced levels of Iba1, P2ry12, Hexb but increased levels in CD11b, whereas some markers (e.g. CX3CR1, Trem2, DAP12 and TLR4) were not changed between the two groups of mice. Lastly and more importantly, our analysis showed a dramatic attenuation in the expression of innate inflammatory cytokines, showing significantly reduced levels of TNFα, IL-1β, IL-10 and TGFβ in LPS challenged APPKO mice compared to BL6. This attenuated cytokine response was even more pronounced in LPS challenged APPKO mice from the older 9 month old cohort (up to ~90% reductions) compared to responses in the younger 3 month old cohort, suggesting an age dependent effect on cytokine expression in response to LPS in APPKO mice. Furthermore, primary microglia cells isolated from APPKO mice and exposed to LPS challenge *in vitro*, were also significantly less responsive to LPS stimulation in terms of expression of Iba1 levels and release of pro-inflammatory cytokines.

Taken together these findings strongly suggest that APP and/or its cleaved products contributes to glia activation in response to acute inflammatory stimuli, to such an extent that in the absence of APP the endogenous innate immune response of the brain is drastically weakened; in particular, microglia phenotype and activation are significantly altered and release of inflammatory cytokines is greatly compromised.

In all of our study groups, we used age-matched uninjected mice as controls for comparison rather than vehicle injected mice. One concern remains as to the potential effects of the injection/surgical procedure itself on the immune response and how it may affect differences observed when comparing the immune response to LPS injection between the treatment groups. Since the magnitude of the immune response to LPS stimulation is rather robust and we see significant differences in the immune response to LPS challenge directly comparing WT BL6 mice vs. APPKO mice, lack of this control group does not appear to influence the overall results and thus interpretation of our data.

Mechanistically, one possible explanation to elucidate the role of APP protein, in the context of the glial activation, is that in order to mount an effective response to injury/inflammation, APP cleaved products (including Aβ peptides and/or sAPPα) transiently up-regulated upon acute brain injury, are directly involved in the activation of glial cells. Indeed, APP fragments, specifically sAPPα, and different conformers of Aβ have been reported to activate microglial cells *in vitro* [17–19], however, our data is the first to demonstrate this microglia specific response *in vivo* in the setting of APPKO mouse background. Similarly, previous reports have shown that APP can confer neuroprotection following brain injury, and that this neuroprotective activity is related to the presence of sAPPα, further supporting a beneficial role of sAPPα in mounting an effective response to injury [16, 20]. Expression of sAPPα alone is able to rescue the abnormalities of APP deficient mice [21], implying that most of APP's physiological function could be mediated by sAPPα alone. Although we have not specifically investigated this role of sAPPα in the present study, it is certainly a focus point for our future investigations.

Alternatively, it is possible that binding and interaction of LPS with APP full length protein on the cell surface may play a role in mounting an effective immune response to inflammatory stimuli. LPS stimulation in cells occurs through a sequence of interactions with many proteins including the LPS binding protein (LBP), CD14, MD-2 and TLR4 [22]. After LPS recognition, TLR4 signaling via NF-κB—MyD88 pathways, then mediates the activation of pro-inflammatory cytokine gene expression. Although APP is generally not thought to act as a “canonical cell surface receptor”, work by Combs et al, [23–25] have documented some evidences for this notion. They have previously reported that APP is associated with a tyrosine kinase-based pro-inflammatory signaling in microglial cells. Both by using β1 integrin-mediated adhesion-dependent activation and cross-linking APP on the cell surface with 22C11,an antibody against the N-terminus of APP, they reported increased protein phosphotyrosine levels in microglia and THP-1 cells, indicative of increased tyrosine kinase activity. In addition to increased tyrosine kinase activity, they also showed activation of the MAP kinase family, and increases in pro-inflammatory markers. Thus one intriguing possibility is that LPS could serve as a ligand for the extracellular domain of APP and this interaction could mediate receptor clustering events and subsequent LPS-TLR dependent signal transduction events, leading to activation of the inflammatory response. Another possibility is that since the intracellular domain (AICD) of APP has been shown to function as a transcription factor [26], modulating genes involved in cell survival and proliferation, it is possible that there is crosstalk with the AICD fragment and LPS-TLR inflammatory signaling pathways. Although our current results suggest a significant contribution of APP on LPS dependent signaling events, future studies will be needed to determine whether LPS can directly or indirectly interact with APP on the cell surface and affect downstream signaling events.

Our studies have highlighted important connection between APP and the innate immune response. As preventative strategies in AD aim to modulate APP processing to lower the Aβ peptide and/or the other specific APP cleaved products with the use of secretase inhibitors, our data support further studies and understanding of the normal physiological functions of APP and its cleaved fragments, particularly with regards to the inflammatory innate immune responses and glial cell responses, to fully evaluate the efficacy and safety of such approaches in the clinic.

## Supporting Information

S1 File Summary of expression levels of mRNAs measured in APPKO and BL6 mice. Expression levels of mRNAs measured in 3 months old APPKO and BL6 mice**(Table A)**. Expression levels of mRNAs measured in 9 months old APPKO and BL6 mice **(Table B)**.
(DOCX)
