# Supplementary material for: Altered Innate Immune and Glial Cell Responses to Inflammatory Stimuli in Amyloid Precursor Protein Knockout Mice
Source: PLoS One. 2015 Oct 8;10(10):e0140210. doi: 10.1371/journal.pone.0140210 (PMC4598170; doi:10.1371/journal.pone.0140210)
Supplement: S1 File — Expression levels of mRNAs measured in 3 months old APPKO and BL6 mice(Table A). Expression levels of mRNAs measured in 9 months old APPKO and BL6 mice (Table B). (DOCX) [file pone.0140210.s001.docx]

| BL6 3 days LPS - APPKO 3 days LPS  3 MONTHS | Mean BL6 | Mean APPKO | Mean Diff. | SE of diff. | % difference | pvalue |
| --- | --- | --- | --- | --- | --- | --- |
|  |  |  |  |  |  |  |
| Iba1 | 17.79 | 7.78 | 10.01 | 5.97 | -56.27 | 0.02 |
| CD11b | 2.46 | 29.68 | -27.22 | 5.97 | 1107.86 | 0.10 |
| CX3CR1 | 3.74 | 2.06 | 1.69 | 6.74 | -45.09 | 0.22 |
| Trem2 | 1.50 | 3.72 | -2.22 | 5.97 | 148.27 | 0.02 |
| DAP12 | 11.62 | 11.64 | -0.02 | 6.74 | 0.18 | 0.99 |
| P2ry12 | 0.47 | 0.33 | 0.14 | 6.39 | -30.58 | 0.31 |
| Hexb | 1.48 | 0.98 | 0.51 | 6.39 | -34.15 | 0.39 |
| TLR4 | 2.38 | 2.64 | -0.27 | 6.15 | 11.15 | 0.80 |
| IL-6 | 5.93 | 6.05 | -0.13 | 2.45 | 2.14 | 0.97 |
| TNFa | 9.48 | 7.19 | 2.28 | 3.62 | -24.08 | 0.19 |
| IL-1b | 8.84 | 2.85 | 5.99 | 3.62 | -67.79 | 0.22 |
| IL-10 | 2.75 | 1.29 | 1.46 | 3.35 | -53.20 | 0.20 |
| TGFb | 1.46 | 0.64 | 0.82 | 3.35 | -56.40 | 0.01 |
| Synaptophysin | 2.60 | 1.86 | 0.74 | 0.35 | -28.54 | 0.05 |
| PSD95 | 3.29 | 1.53 | 1.75 | 0.35 | -53.32 | 0.01 |
| BDNF | 0.54 | 0.88 | -0.34 | 0.35 | 64.13 | 0.13 |
| GFAP | 21.02 | 19.17 | 1.84 | 3.47 | -8.77 | 0.73 |

**Table A.**

| \| BL6 3 days LPS - APPKO 3 days LPS  9 MONTHS \| Mean BL6 \| Mean APPKO \| Mean Diff. \| SE of diff. \| % difference \| pvalue \| \| --- \| --- \| --- \| --- \| --- \| --- \| --- \| \|  \|  \|  \|  \|  \|  \|  \| \| Iba1 \| 10.33 \| 7.71 \| 2.62 \| 1.24 \| -25.35 \| 0.32 \| \| CD11b \| 14.22 \| 16.71 \| -2.49 \| 1.24 \| 17.48 \| 0.12 \| \| CX3CR1 \| 1.94 \| 1.84 \| 0.10 \| 1.18 \| -4.97 \| 0.77 \| \| Trem2 \| 3.54 \| 3.40 \| 0.14 \| 1.24 \| -3.84 \| 0.87 \| \| DAP12 \| 9.39 \| 19.48 \| -10.08 \| 1.45 \| 107.31 \| 0.00 \| \| P2ry12 \| 0.87 \| 0.40 \| 0.47 \| 1.32 \| -54.16 \| 0.01 \| \| Hexb \| 5.54 \| 0.99 \| 4.55 \| 1.32 \| -82.14 \| 0.00 \| \| TLR4 \| 3.15 \| 3.16 \| 0.00 \| 1.32 \| 0.12 \| 0.99 \| \| IL-6 \| 10.36 \| 16.90 \| -6.54 \| 29.99 \| 63.16 \| 0.39 \| \| TNFa \| 113.40 \| 4.84 \| 108.60 \| 28.59 \| -95.77 \| < 0.0001 \| \| IL-1b \| 400.10 \| 63.01 \| 337.10 \| 29.99 \| -84.25 \| 0.00 \| \| IL-10 \| 19.02 \| 1.76 \| 17.26 \| 35.02 \| -90.75 \| 0.02 \| \| TGFb \| 1.01 \| 0.96 \| 0.05 \| 29.99 \| -4.68 \| 0.67 \| \| Synaptophysin \| 0.84 \| 0.94 \| -0.10 \| 0.32 \| 12.14 \| 0.77 \| \| PSD95 \| 0.72 \| 0.89 \| -0.17 \| 0.32 \| 23.97 \| 0.17 \| \| BDNF \| 2.15 \| 3.19 \| -1.03 \| 0.32 \| 47.93 \| 0.05 \| \| GFAP \| 7.67 \| 7.27 \| 0.40 \| 0.75 \| -5.20 \| 0.64 \|   **Table B**. |  |
| --- | --- | --- | --- | --- | --- | --- | --- | --- | --- | --- | --- | --- | --- | --- | --- | --- | --- | --- | --- | --- | --- | --- | --- | --- | --- | --- | --- | --- | --- | --- | --- | --- | --- | --- | --- | --- | --- | --- | --- | --- | --- | --- | --- | --- | --- | --- | --- | --- | --- | --- | --- | --- | --- | --- | --- | --- | --- | --- | --- | --- | --- | --- | --- | --- | --- | --- | --- | --- | --- | --- | --- | --- | --- | --- | --- | --- | --- | --- | --- | --- | --- | --- | --- | --- | --- | --- | --- | --- | --- | --- | --- | --- | --- | --- | --- | --- | --- | --- | --- | --- | --- | --- | --- | --- | --- | --- | --- | --- | --- | --- | --- | --- | --- | --- | --- | --- | --- | --- | --- | --- | --- | --- | --- | --- | --- | --- | --- | --- | --- | --- | --- | --- | --- | --- |
|  |  |
|  |  |
|  |  |
|  |  |
|  |  |
|  |  |
|  |  |
|  |  |
|  |  |
|  |  |
|  |  |
|  |  |
|  |  |
|  |  |
|  |  |
|  |  |
|  |  |
|  |  |
